# A role for cities in sustaining Planetary Health

**DOI:** 10.14324/111.444/ucloe.3601

**Published:** 2026-01-26

**Authors:** Yasemin Didem Aktas, Matthew O. Gribble, Dan Osborn, Lucilla Spini, Pam Berry, Francesco Aletta

**Affiliations:** 1 Department of Civil, Environmental & Geomatic Engineering, University College London, UK; 2 School of Medicine, University of California, San Francisco, USA; 3 Department of Earth Sciences, University College London, UK; 4 Institute for Heritage Science of the Italian National Research Council (CNR-ISPC), Firenze, Italy; 5 Environmental Change Institute, University of Oxford, UK; 6 Bartlett School of Environment, Energy & Resources, University College London, UK

Cities drive planetary change and threaten Planetary Health, but they also can deliver solutions to the environmental crises they help create.

## Dynamic links between humanity and its only planet

The biophysical and human worlds are linked in an interwoven planetary system. People depend on the finite biophysical processes of the Earth for the natural capital and the ecosystem services that support life, society and the economy. The food and water we eat and drink and the air we breathe are direct services received from this system that even provides medicines. Life is indirectly supported through the provision of materials for shelter and energy. Modern urbanised societies rely more on these latter resources than on more rural or traditional ones. For all communities, the way resources are drawn on depends on innovations and choices.

This interwoven planetary and human system has key underpinning components including three biogeochemical cycles – those for carbon, nitrogen and water – and two emergent systems – biodiversity and climate.

Biodiversity, underpinned by the cycling of nutrients, has positive and negative influences on human health and well-being (e.g., organisms that pollinate crops as well as those that carry and cause disease in humans, crops and domestic animals). A stable climate, underpinned by the carbon and water cycles, has been a prerequisite for the development of civilisation during the Holocene.

There is widespread recognition that the crises in the two emergent systems – biodiversity decline and climate – must be addressed urgently. There is growing recognition of a crisis in the hydrological cycle. Thus far, recognition has not been matched by sufficient corrective action with a practicable focus.

In this editorial we set out how such a practicable focus might be contextualised in terms of the dual role that cities may play in helping shape Planetary Health as described by the Rockefeller–*Lancet* Commission [1] and framed as a response to the intensifying trend towards urbanisation.

## Contextualisation

Humans have continuously modified the biophysical world to meet their needs. For example, industrialisation has relied on the modification of the carbon cycle and, in less than 200 years, fossil fuels accumulated over many millions of years have been burned. Likewise, modern agriculture has relied on the modification of the nitrogen cycle at both regional and local scales. The impact of humanity is even evident in accounts of aspects of this cycle that have been stable over geological timescale [2].

These modifications of planetary cycles have brought benefits to many millions of people. However, negative impacts are such that many types of researchers are identifying the limits to the modification of planetary processes. For example, Rockström et al. [3] and Steffen et al. [4] identified nine planetary boundaries which, if crossed, could substantially alter ecosystem functioning and provision of the ecosystem services life depends on. Six [5] and now seven [6] boundaries have recently been assessed as being crossed. Other metrics might usefully define the state of Planetary Health, including components more directly linked to human activity or the economic system such as measures of poverty, deforestation, marine fish catch or land-use change, some of which reflect complexities in the interplay between planetary and human systems [1]. Because planetary systems are non-linear, dynamic and interlinked, Lenton et al. [7] argued that key features of the planetary system could approach ‘tipping points’ and might rapidly transition from one state to some other; a view widely reflected in the literature with much attention and multi-disciplinary debate focused on the strength of the Atlantic Meridional Overturning Circulation [8].

More encouragingly, Maslin [9] and Lenton [10], amongst others, suggest how people might address such crises and even suggest that positive social and market/economic tipping points exist. Recently, Rockström et al. [11] attempted to unite the concept of planetary boundaries and thinking around the “tragedy of the commons”. In doing this, Rockström and colleagues usefully extend the discussion of Planetary Health and forge links to the values that underpin humanity’s culture and heritage, including intangible cultural heritage as defined by the UNESCO 2003 Convention for the Safeguarding of the Intangible Cultural Heritage (https://ich.unesco.org/en/convention and [12]), the economy and livelihoods of many different kinds of communities [13,14].

## Framing

Given the above context it might be argued the central framing question for addressing the crises in climate, biodiversity and water is:

- *To what extent can the biophysical world continue to provide, under a wide range of future scenarios, the services and resources required to maintain human health and thriving human societies whilst not exceeding the planet’s capacity to provide?*

**Figure 1:**
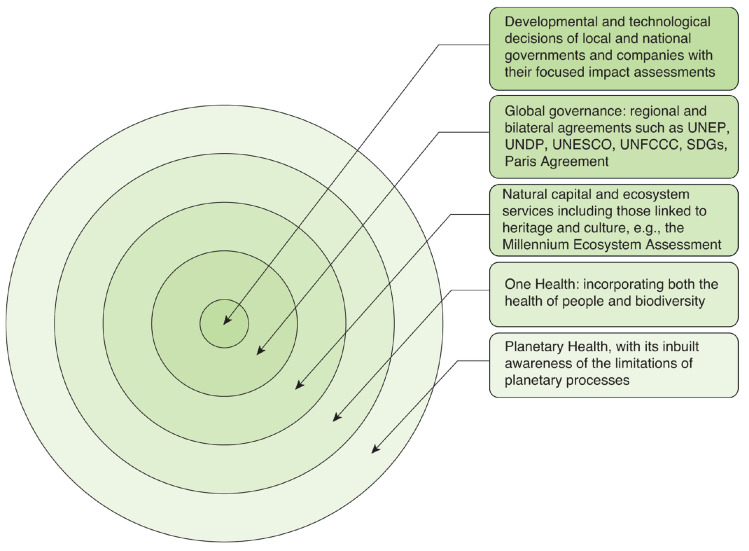
Day-to-day decisions are separated from Planetary Health by a very considerable operational distance that covers substantial temporal and geographical scales.

A key challenge is that the crises humanity faces arise through the cumulative impact of a series of relatively small and often isolated decisions taken by individuals, companies and jurisdictions, all of whom have varying degrees of agency, but none of whom have, or can have, responsibility for the cumulative impacts of their decisions because each one is far removed from the planetary systems that are affected (see Fig. 1). This governance issue, in a sense ‘the tragedy of the commons’ writ large, permits prevarication in decision-making linked to problem solving.

One way to approach dealing with such matters might be to focus on a complex systems area where humanity can and is already exercising control; on an area that is the source of many of the problems humanity currently faces and which already finds practicable solutions that maintain and update systems, *viz* urban areas and, especially perhaps, the large cities that have already been active in international groupings addressing such matters, such as the C40 group of large cities and Local Governments for Sustainability, known as ICLEI (see Box 1).

Box 1. Opening access to research as one approach to crossing the governance divide

Figure 1 illustrates the gaps that exist between isolated decisions and planetary systems. There is also another gap: between research findings and the practical recognition and delivery of evidence-based actions. Various approaches are being taken to address this by research communities and more work like this is likely needed to increase visibility and uptake of evidence. Lowering barriers to publication and opening access to research findings need to be part of this effort. A few examples, all accessed in October 2025, of the many different approaches in use are:

- Policy-relevant semi-technical handbooks such as those that can be found at:
  https://urbanpolicyplatform.org/wp-content/uploads/2021/11/URL-Handbook.pdf
- Gateways to research platforms and co-operative networks as at:
  https://transition-pathways.europa.eu/pse/articles/empowering-rural-and-urban-communities-through-proximity-and-social-economy-ecosystem
  https://iclei.org/
  https://www.c40.org/
  https://www.ucl.ac.uk/bartlett/research-projects/2023/nov/complex-urban-systems-sustainability-and-health-cussh
  https://www.rtpi.org.uk/policy-and-research/practice-and-research/planning-research-matters/resilience-and-adaptation-planning/creating-sustainable-urban-food-water-and-energy-systems/
- Accounts of activities within research groupings or of outreach activities of such groupings:
  https://www.lshtm.ac.uk/research/research-action/features/cities-face-growing-climate-and-health-problems
  https://www.hutton.ac.uk/sites/default/files/files/Rural-and-urban-lessons-learnt.pdf
  https://carey.jhu.edu/articles/healthier-cities-key-healthier-planet-healthier-people
- Regular reports on research findings with particular foci that track change and what this means:
  https://www.planetaryhealthcheck.org/

If the focus were to be cities, then three other framing questions take shape that are relevant and tractable in an urban frame:

- *How much impact can mitigation achieve and how quickly?*
- *What are the limits to adaptation?*
- *What are the likely benefits of mitigation and adaptation in addition over and above the risk reduction that is often difficult to value?*

## The social, economic and environmental importance of cities

Cities have been places of attraction for millennia owing to the security they offer against perils and the riches they offer to those who can grab them. People felt safer closer to many others and enjoyed opportunities not available elsewhere. The rulers of cities also reaped benefits: the City-State of Venice that wielded global and regional influence for centuries, is a case in point.

Gradually, it became clear that people in dense urban areas were more easily exposed to diseases, could more easily be targeted at times of conflict, were at the whim of supply chain problems, and vulnerable to failures of the built environment and infrastructure that could be heightened by natural or manmade hazards, such as earthquakes or fires.

Nevertheless, this has not stopped cities from growing with now 55% of the global population living in them. The global economy is increasingly dominated by the needs (and wants) of the people living in large urban areas. This challenges humanity’s ability to make sustainable decisions, especially given the magnitude of the effort needed to maintain urban services and infrastructures now and in future. For the sheer scale of populations they are home to, the resources needed to support cities and their economic productivity (as currently measured) cities are impressive, despite the fact that they cover on average less than 1% of the Earth’s land surface [15] rising to some 10% or more in densely populated places, exact figures being difficult to obtain [16]. Much of this growth has happened since the early 1990s.

Currently, about 60% of the global economy is linked to cities, with estimates suggesting New York alone will be contributing over £550 billion to global GDP by 2030 [17,18]. Inevitably, this means high energy consumption: over 100,000 TWh annually out of a global total of some 160,000 TWh is directly attributable [19,20]. The share of energy is not distributed evenly between cities of the Global North and South and this is reflected in greenhouse gas emissions [21]. City water consumption is difficult to determine but broadly comes in two parts: freshwater withdrawals of some 10% of the global total can be attributed directly to urban populations, about 380 km^3^ annually. And, although agriculture remains the largest single driver of water withdrawals (some 70–80% of the total), cities are indirectly the main beneficiaries of much of that through food consumption [22,23].

Not all sectors of the economy draw equally on resources or cause emissions correlated with activity due to the varying energy density of fuels (and material equivalents). For example, in 2020, the construction sector (much of which supports transport to and from cities in addition to the buildings and infrastructure actually in cities) emitted about 10% of all energy-related carbon dioxide emissions globally [24]. Following a temporary dip during the Covid-19 pandemic, emissions from the sector rebounded sharply, reaching record highs in 2021 and 2022, driven by increased construction activity, rising housing demand, and supply chain recovery. Building-associated energy consumption remained at 36% of the global demand.

Despite the above issues, cities might not need to cause the indiscriminate consumption of the planet’s resources and services. Cities, by their nature, possess immense potential to drive action on Planetary Health, thanks to their concentrated financial and intellectual capital, leadership, and their spheres of influence. Some cities are already making better use of human capital to make more sustainable use of natural capital. New York City, for instance, avoided constructing a $10 billion water filtration plant by investing in the protection of its Catskill/Delaware watershed, demonstrating how ecosystem services can be harnessed to deliver clean water sustainably [25]. Brisbane has implemented flood-resilient planning through its FloodSmart Future Strategy, which integrates hazard mapping, community engagement and infrastructure adaptation to reduce flood risk while preserving ecological functions [26]. In London, the expansion of the Ultra Low Emission Zone (ULEZ) has significantly reduced nitrogen dioxide and particulate matter levels, improved air quality and reduced health risks [27]. Beijing has pursued aggressive traffic and pollution control measures, including vehicle quotas, promotion of electric vehicles and public transport expansion, resulting in measurable improvements in air quality and public health [28]. These examples demonstrate that cities can actively regenerate rather than deplete natural systems, especially when urban planning is informed by ecological intelligence and inclusive governance. By embedding Planetary Health goals into infrastructure, transport and housing policies, cities can become engines of restoration—supporting biodiversity, reducing emissions and improving public health outcomes simultaneously.

So, are cities a case of a glass half full or half empty in relation to Planetary Health?

## The glass half empty – the impacts of cities on people and the planet

Any change in land use modulates planetary processes at local and regional scales [29]. The extent of the impacts of land-use change is at times unexpected. For instance, land use has been linked to subsurface temperatures [30] with implications for the Earth’s microbial composition, groundwater quality and contaminant behaviour [31]. Harms and damage resulting from land-use change are complex although not necessarily irreversible [32]. Furthermore, urban change is no longer limited to the land. Even the seas are being colonised – as evidenced by the creation of artificial islands and reefs and the deployment of off-shore renewable energy schemes.

To date, urbanisation has been a process that largely sterilises the land, impacts the hydrology of an area and concentrates demand for resources. With urbanisation gaining such remarkable momentum in the last 30 years, cities are becoming focal points for humanity where vulnerabilities to planetary imbalances intensify as the impacts they generate extend far beyond their boundaries.

Interactions between city systems and modified planetary systems impact the health of people and the planet and cause harm [33,34]. Consequent events having negative impacts include flooding, diseases such as cholera, changing local temperature and humidity regimes (manifesting as urban heat/cool and moisture/dry islands), exposure to contaminants which lower air quality, and issues linked to the choices of construction materials, building forms and urban morphologies [35–37].

Poor air quality and excess heat lead to many premature deaths each year. Air pollution leads to the premature death of between 6 and 7 million people annually (see Global Burden of Disease at https://www.thelancet.com/gbd). Many of these are linked to internal and external air quality in large cities and the problem is multifactorial, particularly perhaps in megacities [38]. In addition, the sharp rise in the noise produced by humans has its worst effects in metropolitan areas [39]. In European countries over 20% of the population suffers noise levels affecting their health [40]. Overheating is another cause of mortality [33,41] and the resulting discomfort and injury rates have begun to capture the attention of pressure groups [42].

Impacts on health are not always immediately obvious, and some may originate in the oceans. For example, in the case of tropical cyclones, taking a longer-term and wider geographical perspective shows impacts beyond the immediate physical damage, mortality and resultant mental health impacts of cyclone-associated trauma (such as post-traumatic stress disorder). These lagged effects include: impacts on mortality over a wide area and timespan [43] cancer treatment outcomes arising from interrupted treatments [44] and, achievement in educational tests [45]. Other oceanic impacts linked to public health include issues as different as Ciguatera fish poisoning [46,47] and saltwater intrusion [48] – both varying substantially across geographical locations and community types.

As well as affecting human health, the impact of cities on the planet goes beyond the local and immediate. Ever-growing built-up areas affect water, carbon, nitrogen and aerosol cycles and alter weather–climate systems on wide regional scales through urban forcing [49–51]. This manifests as extreme and atypical weather events and other climatic or climatically triggered geophysical hazards, affecting all aspects of planetary life.

One way cities shape people is to disconnect them from nature. There is evidence of a multi-generational decline in nature connectedness amongst urban people [52]. Such a disconnect suggests an urban, or even metropolitan, culture might be evolving that could, conceivably, reduce people’s ability to make decisions that align with the planet’s resources and renewal processes. City communities and decision-makers might recognise planetary issues but feel powerless to think differently or act either through pressure to keep the existing system running, or lack of knowledge/perceived agency in relation to solutions based on nature or other planetary systems or the influence of misinformation.

Despite all this it could be that cities may also be holding the keys to solving some of humanity’s most pressing problems.

## The glass half full – developing better city systems to address Planetary Health

Cities are not just big towns, they are not just a conglomeration of lots of people and lots of buildings. A city, especially a megacity, is a system of systems, encompassing governance structures, transportation networks, communication infrastructures, economic engines, cultural ecosystems, governance frameworks and environmental dynamics, all woven into an evolving set of entities that shape and are shaped by its people [53]. Such urban process mean cities can be a source of solutions rather than problems, when the nature of cities and the pressures on the communities that live in them are recognised, and efficient governance systems can be established. For example, Rotterdam’s Water Square, Benthemplein, can store up to 1.7 million litres of rainwater during heavy rainfall, reducing flood risk while doubling as a public space when dry. This multifunctional infrastructure is part of a broader strategy that includes underground reservoirs and sponge gardens, which collectively reduce pressure on sewage systems and improve water management [54].

Seoul’s Cheonggyecheon Stream restoration replaced a congested highway with a revitalised urban river, restoring 5.8 km of stream corridor and creating over 276,000 m^2^ of landscaped area. The project led to a 2.3–6°C reduction in ambient temperature and a 35% decrease in particulate matter, significantly improving air quality and reducing urban heat-island effects. Pedestrian activity increased by over 50%, and local business revenues rose by 15–20% [55].

We do not yet know ways of building and running cities that are not at odds with Planetary Health, and we have to work with cities that were founded many decades, even centuries, ago and where political and economic systems are deeply embedded. Even when those responsible for the planning, management and delivery of urban services may be invested in creating urban areas that are better supportive of Planetary Health, the constraints posed by the existing urban infrastructure, organisation and systems may be too restrictive [56–58], making action on behalf of planetary and even human health costly in terms of both economic and social acceptability. However, there are examples which demonstrate that we can surpass these barriers.

Legacy cities like Worcester, Massachusetts and Providence, Rhode Island, have adopted green infrastructure strategies such as bioswales, rain gardens and urban farming. These retrofits have led to reduced flood frequency and improved water quality. Providence’s Climate Justice Plan targets a 30% energy use reduction by 2030 and 100% carbon-free electricity by 2050, with interim goals already showing measurable progress [59].

Cities and large urban areas might also draw inspiration from Indigenous communities and those with more traditional lifestyles linked to the way land or coastal waters are used. Such communities can have much in common with urban communities in terms of the complexities involved in dealing with planetary issues. All communities are experiencing loss of biodiversity and extreme weather. Differing types of approaches to linking urban and other communities have been suggested [60,61]. For example, in Canada, Indigenous-led climate adaptation initiatives have received over $2 billion in federal funding since 2020. Inuit observations of sea ice changes have informed national climate models and policy decisions, while urban Indigenous partnerships are helping cities integrate traditional ecological knowledge into planning and resilience strategies [62].

Thus, cities might learn ways of making decisions more sustainably or acting in relation to Planetary Health issues from smaller or more rural communities whose relations and decisions can be more intimately connected to nature and more easily actionable.

Overall, cities present a conundrum – they are at once vibrant centres of innovation and remarkable engineered systems of systems capable of sustaining the lives of millions of people at high densities, but also, because of the resources such success demands, they are drivers of imbalances in Planetary Health.

The goals for every city seem clear enough: reach net zero by addressing energy use in buildings and transport; deal with flooding of all kinds; minimise pollution of air and water (including by noise); supply, treat and use water sustainably; extend multi-functional green infrastructures; develop ways to limit overheating and the impact of heat islands; foster clean air programmes; minimise waste through engendering a more circular or doughnut economy; reduce inequalities; recognise the importance of maintaining humanity’s culture and heritage including that considered “intangible” (see https://ich.unesco.org/en/convention and [12]). If cities achieved this they would help deliver both the UN Sustainable Development Goals and alleviate current pressures on Planetary Health – but, the extent, nature and impact of this is yet to be fully drawn out.

**Figure 2:**
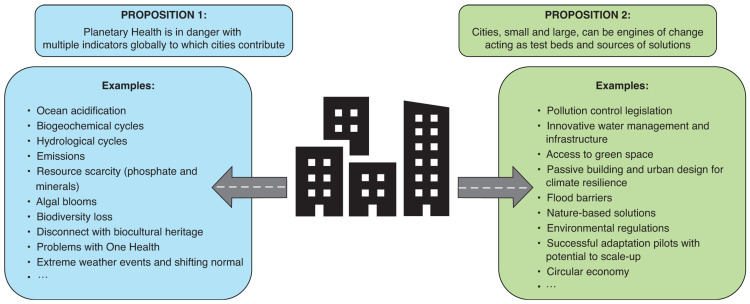
Planetary Health – two propositions.

The planet and its people need a multi-disciplinary and transdisciplinary effort on city systems (as indicated in Fig. 2) to improve Planetary Health and engender the opportunities that could improve human development, make social progress and achieve sustainable economic growth. The damaging effects on planetary systems that are an unintended consequence of the current economic model and current methods of designing and building cities must be constrained so as to keep human activity within planetary limits. Approaches to the complexity involved are already being suggested [63].

Any multidisciplinary and/or transdisciplinary effort will have the best chance of success and impact if knowledge is shared openly and at reasonable cost. This means research findings, reviews of various kinds and evidence-based commentary and policy relevant reports should be available as widely and as freely as possible. This is why the journal’s Editorial Board feels the journal is a well-placed to make a contribution to Planetary Health by hosting part of the necessary discourse. Submissions are thus invited on any topic related to the issues this editorial raises. Calls for submissions in particular areas of interest will appear soon.
